# The first 2 years of COVID-19 in Italy: Incidence, lethality, and health policies

**DOI:** 10.3389/fpubh.2022.986743

**Published:** 2022-11-01

**Authors:** Pierpaolo Ferrante

**Affiliations:** Department of Occupational and Environmental Medicine, Epidemiology and Hygiene, Italian National Workers' Compensation Authority (INAIL), Rome, Italy

**Keywords:** COVID-19, incidence, lethality, health policy, negative binomial, moving averages

## Abstract

**Background:**

The novel coronavirus disease 2019 (COVID-19) is an ongoing pandemic that was first recognized in China in December 2019. This paper aims to provide a detailed overview of the first 2 years of the pandemic in Italy.

**Design and methods:**

Using the negative binomial distribution, the daily incidence of infections was estimated through the virus's lethality and the moving-averaged deaths. The lethality of the original strain (estimated through national sero-surveys) was adjusted daily for age of infections, hazard ratios of virus variants, and the cumulative distribution of vaccinated individuals.

**Results:**

From February 24, 2020, to February 28, 2022, there were 20,833,018 (20,728,924–20,937,375) cases distributed over five waves. The overall lethality rate was 0.73%, but daily it ranged from 2.78% (in the first wave) to 0.15% (in the last wave). The first two waves had the highest number of daily deaths (about 710) and the last wave showed the highest peak of daily infections (220,487). Restriction measures of population mobility strongly slowed the viral spread. During the 2nd year of the pandemic, vaccines prevented 10,000,000 infections and 115,000 deaths.

**Conclusion:**

Almost 40% of COVID-19 infections have gone undetected and they were mostly concentrated in the first year of the pandemic. From the second year, a massive test campaign made it possible to detect more asymptomatic cases, especially among the youngest. Mobility restriction measures were an effective suppression strategy while distance learning and smart working were effective mitigation strategies. Despite the variants of concern, vaccines strongly reduced the pandemic impact on the healthcare system avoiding strong restriction measures.

## Introduction

The severe acute respiratory syndrome coronavirus 2 (SARS-CoV-2) is a new virus identified in Wuhan (Hubei, China) in late 2019 (1). SARS-CoV-2 causes the coronavirus disease 2019 (COVID-19), an illness that ranges from mild flu symptoms to bilateral interstitial pneumonia (2). The virus spread so quickly around the world that the World Health Organization (WHO) declared the COVID-19 a Public Health Emergency of International Concern on January 30, 2020 and a pandemic on March 11, 2020, (3). Unlike other coronaviruses, the SARS-CoV-2 is able to spread through pre- and asymptomatic infections that are difficult to detect and isolate, requiring health authorities to test all contacts of confirmed cases to lower the risk of spread (4, 5). The lethality of the original strain was estimated using infection fatality ratios (IFR) assessed through several national sero-surveys (6, 7). While relatively low in the whole population (< 1 death per 100 infections in developed countries), the risk of death is shown to increase with age (up to 10–15 deaths per 100 infections in people aged more than 75 years) and in patients who are immunosuppressed or have concomitant comorbidities (8, 9). Furthermore, since the prognosis of severe cases depends on the availability of intensive care beds, lethality increases when critical care capacity is saturated (10). To address the pandemic, a global vaccination campaign was launched, and pharmaceutical industry developed candidate COVID-19 vaccines at an unprecedented speed. By the end of 2020, global Medicines Agencies had conditionally approved several vaccines based on different technologies, with others close behind (11, 12). During the first 2 years of the pandemic (since December 29, 2019, to February 28, 2022), National Health Institutions detected 444,900,763 confirmed cases and reported 6,020,752 deaths worldwide^1^. The highest number of infections favored mutations in the viral genome sequence and led to generation and spread of many viral variants (13). WHO coordinates national and subnational research aimed at sequencing RNA viral genomes detected in infected people and classifying variants of concern (VOC) that may pose a greater risk to global public health^2^. From May 2020 to February 2022, WHO identified five consecutive VOCs: Alpha, Beta, Gamma, Delta, and Omicron. Each variant showed an increased capacity to spread (even within vaccinated people) and although the debate on virulence is still open, it would appear that all the VOCs except Omicron caused a disease with higher severity and mortality (14, 15). In February 2020, Italy was the European epicenter of the SARS-CoV-2 spreading. The unexpectedly high speed of transmissions quickly resulted in hospital saturation and forced the Italian government to establish a national lockdown. Restriction measures blocked the first wave and were gradually removed in parallel with the development of a robust COVID-19 contact tracing system. To avoid lockdown during the second wave, the national government has applied a standard set of restriction measures (from soft to hard) at the regional level based on the risk of spread evaluated on a weekly basis. The risk level by geographic area (represented by a colored map: white = low, yellow = moderate, orange = high, red = highest) was evaluated by determining weekly estimates of incidence and reproductive number (*R*_*t*_). During the second wave (December 27th, 2020), a national vaccination campaign was launched using two messenger RNA (Pfizer-BioNTech, Moderna) and two vector vaccines (Janssen, Vaxzevria) (14). Given the high percentage of vaccinated people in the third wave, the hospital saturation levels replaced the *R*_*t*_ in the risk evaluation. Although vaccine protection declined over time (especially against virus variants), protection returned following administration of the booster dose especially against the development of severe infections (16–19). Health institution recommended a booster shot after 4 months from the standard cycle in September 2021, and included children aged 5–11 years in the vaccine campaign in December 2021 (19, 20). This study aims to provide a detailed overview of the first 2 years of the pandemic in Italy, where 13,000,000 of confirmed cases and 155,000 deaths were reported from February 2020 to 2022. The current paper is part of a larger project aimed at describing the epidemiology of Italian COVID-19 pandemic and follows an initial article introducing the method used to describe the pandemic in its 1st year (21).

## Methods

### Study design

This study analyzed public data of COVID-19 in Italy collected in the national registry by the Civil Protection (CP) and the National Health Institute (ISS).

### Settings

The Italian Government declared a health emergency status on January 3, 2020 and extended it to March 31, 2022. The CP was delegated to manage the process and established a system to collect COVID-19 data in a national registry (managed by the ISS). Aggregate data on incidence and vaccination are published daily. The ISS reviews and updates the registry data to account for data reporting delays and regional recounts and releases an updated report with details including the age distribution of detected cases. The European Center for Disease Prevention and Control (ECDC) collects VOCs continental data through the European Surveillance System (TESSy).

### Participants

All confirmed cases of COVID-19 in Italy.

### Outcomes

The primary outcomes were: (1) the number **N**_**k**_ of persons who became infected on the *k*th day of pandemic; (2) the number **D**_**k**_ of persons who died (over time) among **N**_**k**_ (i.e., the number of deaths by the infection day); (3) the number **υ**_**k**_ of persons who were officially detected among **N**_**k**_ (i.e., the number of diagnosed cases by the infection day).

### Data sources/measurement

Aggregate data from the national COVID-19 registry and the vaccine campaign are stored in public repositories and updated daily. The data include daily counts of performed tests, of diagnosed cases and fatalities who tested positive using the polymerase chain reaction or the rapid antigen test (beginning on January 8, 2021), and of persons who received vaccine shots by region^3^. The ISS provides a weekly report that includes the median age of detected cases, estimates of vaccine protection and (beginning on December 7, 2020) the distribution of detected cases by 10-year age class^4^ (22). The ECDC releases European data on VOCs^5^, the National Institute of Statistics releases data from the sero-survey (May 25–July 15, 2020)^6^ and on Italian population^7^.

### Statistical analysis

As already highlighted by De Natale et al. at the onset of the pandemic, the high number of asymptomatic infections makes deaths more suitable than detected cases for estimating incidence (23). Given the probability *p*_*k*_ of dying after having caught the infection on the *k*th pandemic day (*k*∈ℤ^+^), we used the negative binomial distribution to estimate the daily number of infections (*N*_*k*_) from the resulting deaths over time [*D*_*k*_; Section Modeling the Incidence of Infections (Negative Binomial Distribution)]. First, we estimated *D*_*k*_ by applying the weighted moving average to deaths (that are recorded by the occurrence date, Section Estimating *D*_*k*_ and υ_*k*_: Weighted Moving Average). Second, we modeled the probability *p*_*k*_ accounting for the age at infection, VOCs prevalence and population vaccination level (Section Modeling the Daily Probability to Die *p*_*k*_). Using other simple assumptions, we evaluated excess death (for health system saturation) and lives saved by vaccines (Sections Excess Death and Vaccine Effect). Finally, we used the number of detected cases υ_*k*_ among *N*_*k*_ to check the admissibility (*N*_*k*_>υ_*k*_) of estimates (Sections Estimating *D*_*k*_ and υ_*k*_: Weighted Moving Average and Checking Estimates). In the following, we will proceed with the mathematical formulation, which will be progressively upgraded, in the next sections, to consider the more complex probabilities involving age classes, different strains, and vaccination level. Once the main formulas are established, the estimated variables used to determine the solutions will be given in the Section Estimating the Daily Lethality.

#### Modeling the incidence of infections (negative binomial distribution)

Let $X_{k}^{\left(j\right)}$ be the binary random variable representing the outcome (1 = dead; 0 = recovered) of the *j*th person infected on the *k*th day of the pandemic


$$X_{k}^{\left(j\right)}=\{\;\begin{array}{c} \;1\;p_{k}\;\\ \;0\;1-p_{k} \end{array}$$

and let $N_{k}^{\left(D_{k}\right)}$ be the random variable representing the rank of the daily infection resulting in the *D*_*k*_-th death, the probability of $N_{k}^{\left(D_{k}\right)}$ follows a negative binomial distribution with parameters *D*_*k*_ and *p*_*k*_


(1) $$\begin{array}{l} P\left\{N_{k}^{\left(D_{k}\right)}=n\right\}=P\{\sum_{j=1}^{n-1}X_{k}^{\left(j\right)}=D_{k}-1,\\ \;\sum_{j=1}^{n}X_{k}^{\left(j\right)}=D_{k}\}\\ \;=\left(\begin{array}{c} n-1\\ D_{k}-1 \end{array}\right)p_{k}^{D_{k}}{\left(1-p_{k}\right)}^{n-D_{k}} \end{array}$$

with $k,D_{k}\in Z^{+},n\geq D_{k}$. We estimated the number of daily infections (with the related 95% CI) as the mean of Equation (1)


(2) $$\begin{array}{l} {\hat{N}}_{k}^{\left(D_{k}\right)}=E\left[N_{k}^{\left(D_{k}\right)}\right]=\frac{D_{k}}{p_{k}}. \end{array}$$

#### Estimating D_k_ and υ_k_: Weighted moving average

Let *d*_*k, k*+*j*_ and υ_*k, k*+*j*_ be the number of persons infected on the *k*th pandemic day who died or were diagnosed *j* days after the infection, the number of deaths (*D*_*k*_), and detected cases (*V*_*k*_) among infections on the *k*th pandemic day can be evaluated as


$$\begin{array}{l} D_{k}=\sum_{j}d_{k,k+j}\text{ and }V_{k}=\underset{j}{\sum }\upsilon_{k,k+j}. \end{array}$$

Since only the corresponding number of events by the occurrence date (of death or diagnosis) is available


(3) $$\begin{array}{l} d_{\cdot ,k+j}=\sum_{i}d_{i,k+j}\text{ and }\upsilon_{\cdot ,k+j}=\sum_{i}\upsilon_{i,k+j} \end{array}$$

*D*_*k*_ and *V*_*k*_ were estimated as


(4) $$\begin{array}{l} D_{k}=\sum_{j}\pi_{j}^{\left(k+j\right)}d_{\cdot ,k+j}\text{ and }V_{k}=\sum_{j}\theta_{j}^{\left(k+j\right)}\upsilon_{\cdot ,k+j}, \end{array}$$

where $\pi_{j}^{\left(k+j\right)}$ and $\theta_{j}^{\left(k+j\right)}$ are the fractions


$$\begin{array}{l} \pi_{j}^{\left(k+j\right)}=\frac{d_{k,k+j}}{d_{\cdot ,k+j}}\text{ and }\theta_{j}^{\left(k+j\right)}=\frac{\upsilon_{k,k+j}}{\upsilon_{\cdot ,k+j}}. \end{array}$$

Let *T*_*dead*_ and *T*_*diagn*_ represent the time from infection to death and diagnosis, respectively, and α_*k*_ and β_*k*_ be the binary variables representing the events to die (α_*k*_ = 1) or be alive (α_*k*_ = 0) and to be diagnosed (β_*k*_ = 1) or undetected (β_*k*_ = 0) on the *k*th pandemic day, $\pi_{j}^{\left(k+j\right)}$ and $\theta_{j}^{\left(k+j\right)}$ can be expressed as the conditional probability to die or be diagnosed *j* days after the infection


(5) $$\begin{array}{l} \pi_{j}^{\left(k+j\right)}=P\{j\leq T_{dead}<j+1|\alpha_{k+j}=1\}\text{ and }\\ \theta_{j}^{\left(k+j\right)}=P\{j\leq T_{diagn}<j+1|\beta_{k+j}=1\}. \end{array}$$

The ISS provided estimated quartiles (*Q*1, *Q*2, and *Q*3) of the time distributions from symptoms to death and diagnosis during three different periods (March-May/2020, June-September/2020, and October/2020-December/2020). The ISS estimates for time to death are admissible under symmetric distributions except during the summer period [where there was strong bias from clusters of vacationers (24)]. These biased estimates were not considered and the remaining, which are equivalent [Table 1 in (21)], were extended to the whole studied period. We added 5 days [the mean time from infection to symptoms (25)] to ISS estimates to obtain the corresponding parameters of the probability density function of the time from infection to death and diagnosis


(6) $$\begin{array}{l} f_{T_{dead}}^{\left(\alpha_{k}=1\right)}\left(t\right)=\frac{d}{dt}P\{T_{dead}<t|\alpha_{k}=1\}\text{ and}\\ f_{T_{diagn}}^{\left(\beta_{k}=1\right)}\left(t\right)=\frac{d}{dt}P\{T_{diagn}<t|\beta_{k}=1\}. \end{array}$$

If necessary, we adjusted for symmetry by replacing the median with the center of first and third quartile and assumed that the functions in Equation (6) follow the truncated normal distribution


(7) $$\begin{array}{l} F_{T}\left(t\right)=\frac{\frac{e^{-\frac{1}{2}{\left(\frac{t-\mu }{\sigma }\right)}^{2}}}{\sigma \sqrt{2\pi }}}{\int_{0}^{2\mu }\;\frac{e^{-\frac{1}{2}{\left(\frac{t-\mu }{\sigma }\right)}^{2}}}{\sigma \sqrt{2\pi }}\;dt}\text{ with}t\in \left[0,2\mu \right], \end{array}$$

where μ and σ are the mean and standard deviation of the parent general normal probability with $\mu =\frac{Q_{3}+Q_{1}}{2}$ and $\sigma =\frac{Q_{3}-Q_{1}}{1.34896}$. Of note, the Equation (4) with probabilities Equation (5) derived from Equation (7) can be also interpreted as a weighted moving average of period 2μ+1 on time series *d*_., *k*+*j*_ and υ_., *k*+*j*_ in (3)


$$\begin{array}{l} D_{k}=\sum_{j=0}^{2\mu }\pi_{j}^{\left(k+j\right)}d_{.,k+j}\text{ and }V_{k}=\sum_{j=0}^{2\mu }\theta_{j}^{\left(k+j\right)}\upsilon_{.,k+j}. \end{array}$$

#### Modeling the daily probability to die p_k_

Let *X*_*j*, ξ, *V*_ and *Y*_*k, j*, ξ, *V*_ be the binary random variables representing the events “to die after the infection” and “to be infected on the *k*th pandemic day,” respectively, by 10-year age class (*j*: 0–9, 10–19, …, 80–89, 90+ years), VOC (ξ: 0 = original strain; 1 = Alpha; 2 = Beta; 3 = Gamma;4 = Delta; 5 = Omicron), and vaccination level (*V*: 0 = unvaccinated; 1 = uncompleted basic cycle; 2 = completed basic cycle more than 4 months ago; 3 = completed basic cycle in the last 4 months; 4 = received a booster shot). By assuming that the conditional probability *p*_*k, j*, ξ, *V*_ to die after having caught the infection on the *k*th day does not depend on *k*, we have that


(8) $$\begin{array}{l} p_{j,\xi ,V}=P\{X_{j,\xi ,V}=1|Y_{k,j,\xi ,V}=1\}\;\forall \;k\in {\mathbb{Z}}^{+}. \end{array}$$

Let *N*_*k, j*, ξ, *V*_ be the number of infected people on the *k*th pandemic day by age class, VOC, and vaccination level and *N*_*k*, ., ., ._ be the total number of infections on the same day, the overall probability *p*_*k*_ to die among infections on the *k*-th day is equal to


(9) $$\begin{array}{l} p_{k}=\sum_{j}\sum_{\xi }\sum_{V}p_{j,\xi ,V}\frac{N_{k,j,\xi ,V}}{N_{k,.,.,.}}. \end{array}$$

Now, let *RR*_*j*, ξ, *V*_ be the risk ratio to die of people with the vaccination level *V* (= 0, 1, 2, 3, 4) vs. unvaccinated (*V* = 0) by age class and VOC


(10) $$\begin{array}{l} RR_{j,\xi ,V}=\frac{p_{j,\xi ,V}}{p_{j,\xi ,0}}, \end{array}$$

and let *N*_*k, j*, ξ, ._ and *N*_*k, j*, ., ._ be the number of infections on the *k*th pandemic, respectively, by age class and VOC (with any vaccination level) and by age class (with any VOC and vaccination level), the Equation (9) can be rewritten through the Equation (10) as


(11) $$\begin{array}{l} p_{k}=\sum_{j}\left[\sum_{\xi }\left(\sum_{V}RR_{j,\xi ,V}\frac{N_{k,j,\xi ,V}}{N_{k,j,\xi ,.}}\right)p_{j,\xi ,0}\frac{N_{k,j,\xi ,.}}{N_{k,j,.,.}}\right]\frac{N_{k,j,.,.}}{N_{k,.,.,.}}. \end{array}$$

Finally, let *S*_*j*, ξ, 0_(*t*) and *S*_*j*, 0, 0_(*t*) be the distributions of survival time of unvaccinated people in the *j*th age class, respectively, for the VOC ξ (= 0, 1, 2, 3, 4, 5) and original virus strain (ξ = 0), under the assumption of proportional hazards we have that


(12) $$\begin{array}{l} {\frac{d}{dt}log\left[S_{j,\xi ,0}\left(t\right)\right]=hR}_{j,\xi ,0}\frac{d}{dt}log\left[S_{j,0,0}\left(t\right)\right], \end{array}$$

where *hR*_*j*, ξ, 0_ are the hazard ratios by VOC (ξ = 0, 1, 2, 3, 4, 5 vs. ξ = 0) by age class for unvaccinated people (*V* = 0). By integrating the Equation (12) over the whole pandemic period, we obtain the following identity


(13) $$\begin{array}{l} S_{j,\xi ,0}={\left[S_{j,0,0}\right]}^{hR_{j,\xi ,0}} \end{array}$$

and since *S*_*j*, ξ, 0_ = 1−*p*_*j*, ξ, 0_ and *S*_*j*, 0, 0_ = 1−*p*_*j*, 0, 0_ the Equation (11) can be expressed as


(14) $$\begin{array}{l} p_{k}=\sum_{j}\{\sum_{\xi }\left(\sum_{V}RR_{j,\xi ,V}\frac{N_{k,j,\xi ,V}}{N_{k,j,\xi ,.}}\right)\\ \;[1-{\left(1-p_{j,0,0}\right)}^{hR_{j,\xi ,0}}]\frac{N_{k,j,\xi ,.}}{N_{k,j,.,.}}\}\frac{N_{k,j,.,.}}{N_{k,.,.,.}}. \end{array}$$

The Equation (14) is the lethality equation I introduced to compute the pandemic parameters of interest. We can notice that it depends on *k* only through the daily distribution of infection by age, VOC, and vaccination level and that we can derive the lethality by variant as


$$\begin{array}{l} p_{k,\xi }=\sum_{j}\left(\sum_{V}RR_{j,\xi ,V}\frac{N_{k,j,\xi ,V}}{N_{k,j,\xi ,.}}\right)\\ \;[1-{\left(1-p_{j,0,0}\right)}^{hR_{j,\xi ,0}}\;]\frac{N_{k,j,\xi ,.}}{N_{k,.,\xi ,.}}, \end{array}$$

where $\frac{N_{k,j,\xi ,.}}{N_{k,.,\xi ,.}}$ is the age distribution of infections due to the variant ξ. All still unknown quantities used to univocally determine the result will be specified in the paragraph Estimating the daily lethality.

#### Excess death

Let *P* and *P*_*j*_ be, respectively, the Italian population and its subgroup in the *j*-th age class and $Y_{i}^{\left(j\right)}$ be the binary random variable indicating that the *i*th person in the *j*th age class has been infected. If the virus spreads randomly within the population,


$$\begin{array}{l} P\{Y_{i}^{\left(j\right)}=1\}=\frac{1}{P}\forall i,j \end{array}$$

we would have that the distribution of cases by age class equals that of the whole population


(15) $$\begin{array}{l} \sum_{i=1}^{P_{j}}P\{Y_{i}^{\left(j\right)}=1\}=\frac{P_{j}}{P} \end{array}$$

and the related probability of dying can be obtained from Equation (14) by replacing the proportion of infected people by age class ($\frac{N_{k,j,.,.}}{N_{k,.,.,.}}$) with the corresponding proportion in the population ($\frac{P_{j}}{P}$),


(16) $$\begin{array}{l} p_{k}^{+}=\sum_{j}\{\sum_{\xi }\left(\sum_{V}RR_{j,\xi ,V}\frac{N_{k,j,\xi ,V}}{N_{k,j,\xi ,.}}\right)\\ \;[1-{\left(1-p_{j,0,0}\right)}^{hR_{j,\xi ,0}}]\frac{N_{k,j,\xi ,.}}{N_{k,j,.,.}}\}\frac{P_{j}}{P}. \end{array}$$

Since COVID-19 transmission began among younger people and eventually spread within the elderly (26), it was assumed that the spread was out-of-control if the distribution of detected cases by age class followed the age structure of the population (15). Through the deaths that resulted from the product between the Equation (2) and the Equation (16),


$$\begin{array}{l} D_{k}^{+}={\hat{N}}_{k}^{\left(D_{k}\right)}p_{k}^{+}=\;\frac{{\hat{D}}_{k}}{p_{k}}p_{k}^{+}, \end{array}$$

the excess death was defined as the following difference:


$$\begin{array}{l} D_{k}^{Excess}=D_{k}^{+}-\;{\hat{D}}_{k}. \end{array}$$

#### Vaccine effect

##### Avoided infections

By rewriting the conditional probability in Equation (8) as ratio of probabilities, the relative risk in Equation (10) can be expressed as


(17) $$\begin{array}{l} RR_{j,\xi ,V}=\frac{P\{X_{j,\xi ,V}=1,Y_{k,j,\xi ,V}=1\}/P\{Y_{k,j,\xi ,V}=1\}}{P\{X_{j,\xi ,0}=1,Y_{k,j,\xi ,0}=1\}/P\{Y_{k,j,\xi ,0}=1\}} \end{array}$$

Under the assumption that the vaccines had no impact on the risk of catching the infection (*P*{*Y*_*k, j*, ξ, *V*_ = 1} = *P*{*Y*_*k, j*, ξ, 0_ = 1}), the relative risk (17) reduces to:


(18) $$\begin{array}{l} RR_{j,\xi ,V}^{\left(.,0\right)}=\frac{P\{X_{k,j,\xi ,V}=1,Y_{k,j,\xi ,V}=1\}}{P\{X_{k,j,\xi ,0}=1,Y_{k,j,\xi ,0}=1\}}. \end{array}$$

Finally, let *Pop*_*k, j*, ξ, *V*_ be the population at risk on the *k*th pandemic by age class, VOC, and vaccination level and *D*_*k, j*, ξ, *V*_ be the number of deaths in each group, through the relationship


$$\begin{array}{l} D_{k,j,\xi ,V}=\;P\{X_{j,\xi ,V}=1,Y_{k,j,\xi ,V}=1\;\}Pop_{k,j,\xi ,V}, \end{array}$$

the relative risk (18) can be rewritten as


(19) $$\begin{array}{l} RR_{j,\xi ,V}^{\left(.,0\right)}=\frac{D_{k,j,\xi ,V}/Pop_{k,j,\xi ,V}}{D_{k,j,\xi ,0}/Pop_{k,j,\xi ,0}} \end{array}$$

By replacing in Equation (14) *RR*_*j*, ξ, *V*_ with $RR_{j,\xi ,V}^{\left(.,0\right)}$, we obtain the daily probability $p_{k}^{\left(.,0\right)}$ to die if the vaccines have no protective effects against catching infection. By replacing in Equation (2) *p*_*k*_ with $p_{k}^{\left(.,0\right)}$, we can estimate the number of infections that would have occurred without vaccines


(20) $$\begin{array}{l} {\hat{N}}_{k}^{\left(0\right)}=\frac{D_{k}}{p_{k}^{\left(.,0\right)}}. \end{array}$$

##### Saved lives

If the vaccines have no effect against death, the relative risks *RR*_*j*, ξ, *V*_ in Equation (14) would be equal to 1 and the lethality would reduce to


(21) $$\begin{array}{l} p_{k}^{\left(0,.\right)}=\sum_{j}\{\sum_{\xi }\left[1-{\left(1-p_{j,0,0}\right)}^{hR_{j,\xi ,0}}\right]\frac{N_{k,j,\xi ,.}}{N_{k,j,.,.}}\}\frac{N_{k,j,.,.}}{N_{k,.,.,.}}. \end{array}$$

By multiplying the Equation (20) for the Equation (21), we obtain an estimate of the number of deaths $D_{k}^{**}$ that would have occurred without vaccines


$$\begin{array}{l} D_{k}^{**}={\hat{N}}_{k}^{\left(0\right)}p_{k}^{\left(0,.\;\right)}. \end{array}$$

By multiplying the Equation (2) for the Equation (21), we obtain an estimate of the number of deaths $D_{k}^{*}$ that would have occurred without vaccines among the infected people


$$\begin{array}{l} D_{k}^{*}={\hat{N}}_{k}p_{k}^{\left(0,.\;\right)}. \end{array}$$

#### Checking estimates

We studied the ratios ${\hat{r}}_{k}$ of detected cases ${\hat{\upsilon }}_{k}$ among estimated infections ${\hat{N}}_{k}$ on *k*th day (Figure 2)


$$\begin{array}{l} {\hat{r}}_{k}=\frac{{\hat{\upsilon }}_{k}}{\;{\hat{N}}_{k}}. \end{array}$$

If ${\hat{r}}_{k}\left(i\right)>$1 (i.e., ${\hat{\upsilon }}_{k}>{\hat{N}}_{k}$), then the estimated ${\hat{p}}_{k}$ overestimates the actual *p*_*k*_ on the *k*th pandemic day


$$\begin{array}{l} p_{k}<\;{\hat{p}}_{k}. \end{array}$$

#### Estimating the daily lethality

Available data to estimate quantities in Equation (14) were used as follows:

1) The probability *p*_*j*, 0, 0_ of dying among unvaccinated (*V* = 0) people in the *j*th age class who were infected with the original strain (ξ = 0) was estimated using the IFR by age class (${\hat{IFR}}_{j,0,0}$) in (6)

(22) $$\begin{array}{l} {\hat{p}}_{j,0,0}={\hat{IFR}}_{j,0,0}. \end{array}$$

2) The daily unvaccinated population by age (*N*_*k, j*, ., 0_) was estimated as the difference between the ISTAT population^7^ and the vaccinated people^4^.
3) As estimates of hazard ratios *hR*_*j*, ξ, 0_ were used those from (27, 28) and since those for Alpha, Beta, Gamma, and Delta are not determined by age class, we considered them constant by age.
4) Let *D*_*j*, ξ, *V*_, *C*_*j*, ξ, *V*_, and *Pop*_*j*, ξ, *V*_ be the number of deaths, of detected infections, and of population by age class, VOC, and vaccination level, respectively. The ISS provided estimates of the relative rate (vaccinated/unvaccinated) of deaths (*RD*_*j*, ξ, *V*_) and of infections (*RC*_*j*, ξ, *V*_)
$$\begin{array}{l} {\hat{RD}}_{j,\xi ,V}=\frac{{\hat{D}}_{j,\xi ,V}/{\hat{Pop}}_{j,\xi ,V}}{{\hat{D}}_{j,\xi ,0}/{\hat{Pop}}_{j,\xi ,0}}\text{ and }{\hat{RC}}_{j,\xi ,V}\;=\;\frac{\frac{{\hat{C}}_{j,\xi ,V}}{{\hat{Pop}}_{j,\xi ,V}}}{\frac{{\hat{C}}_{j,\xi ,0}}{{\hat{Pop}}_{j,\xi ,0}}}, \end{array}$$
  for the periods January–September/2021, October/2021, November/2021, December/2022, January /2022, and February/2022 (22). We used those estimates to assess the relative risk in Equation (10) and in Equation (19) as follows

(23) $$\begin{array}{l} {\hat{RR}}_{j,\xi ,V}=\frac{{\hat{RD}}_{j,\xi ,V}}{{\hat{RC}}_{j,\xi ,V}}=\frac{{\hat{D}}_{j,\xi ,V}/{\hat{C}}_{j,\xi ,V}}{{\hat{D}}_{j,\xi ,0}/{\hat{C}}_{j,\xi ,0}}\text{ and}\\ {\hat{RR}}_{j,\xi ,V}^{\left(.,0\right)}=\;\frac{{\hat{RD}}_{j,\xi ,V}}{{\hat{RC}}_{j,\xi ,0}}={\hat{RD}}_{j,\xi ,V}. \end{array}$$

5) As estimates of the fraction of vaccinated infections by age class and VOC ($\frac{N_{k,j,\xi ,V}}{N_{k,j,\xi ,.}}$), the daily fraction of vaccinated population by age class^4^ were used. The more a VOC is prevalent, the lesser the introduced bias.
6) As daily fraction of infections for each VOC (ξ = 0, 1, …, 5) by age class ($\frac{N_{k,j,\xi ,.}}{N_{k,j,.,.}}$) daily estimates from^5^ were used.
7) As estimates of age distribution of total infections ($\frac{N_{k,j,.,.}}{N_{k,.,.,.}}$) and of those by VOC ($\frac{N_{k,j,\xi ,.}}{N_{k,.,\xi ,.}}$), was used the daily age distribution of detected cases released by the ISS from December 8, 2020 ($f_{k,j}^{\left(ISS\right)}$)^3^. For the precedent period (during which the ISS only released the median age of detected cases), we constructed fictitious populations $P^{\left(Med_{k}\right)}$ with median ages (*MED*s) equal to those estimated and with the age structure related with that of Italian population provided by the ISTAT^7^ [Section Estimating $f_{j,k}^{\left(Med_{k}\right)}$].

##### Estimating $f_{j,k}^{\left(Med_{k}\right)}$

Let *P*_*j*_ and $P_{k,j}^{\left(Med_{k}\right)}$ be the people of age *j* in the Italian and in fictitious populations, respectively, on the *k*th pandemic day, using the definition of “median” we have that


(24) $$\begin{array}{l} \;P_{k,0}^{\left(Med_{k}\right)}\sum_{j=0}^{Med_{k}}\frac{P_{k,j}^{\left(Med_{k}\right)}}{P_{k,0}^{\left(Med_{k}\right)}}=0.5\text{ and }\\ P_{k,100+}^{\left(Med_{k}\right)}\sum_{j=Med_{k}+1}^{100}\frac{P_{k,j}^{\left(Med_{k}\right)}}{P_{k,100+}^{\left(Med_{k}\right)}}=0.5, \end{array}$$

where 100+ indicate people aged 100 years and more. By assuming that the ratios between infected people at ages greater than the median and the oldest (100+ years) are equal to those in the Italian population, and that the ratios between those at ages smaller than or equal to the median and the youngest (0 years) are also equal to those in the Italian population


(25) $$\begin{array}{l} \frac{P_{k,j}^{\left(Med_{k}\right)}}{P_{k,0}^{\left(Med_{k}\right)}}=\frac{P_{j}}{P_{0}}\text{with }\text{j}=0,1,\ldots ,\text{MED and}\\ \frac{P_{k,j}^{\left(Med_{k}\right)}}{P_{k,100+}^{\left(Med_{k}\right)}}=\frac{P_{j}}{P_{100+}}\text{with }\text{j}=\text{MED+1, …, 100+}, \end{array}$$

$P_{k,0}^{\left(Med_{k}\right)}$ and $P_{k,100+}^{\left(Med_{k}\right)}$ are determined from the Equation (24) and can be used to derive all the remaining fractions ($f_{k,j}^{\left(Med_{k}\;\right)}$)


$$\begin{array}{l} f_{k,j}^{\left(Med_{k}\right)}=\frac{P_{k,j}^{\left(Med_{k}\right)}}{P_{k,.}^{\left(Med_{k}\right)}}\;\text{j}=1,\ldots ,\;100+. \end{array}$$

Since the method returns one probability estimate per week (ISS median age refers to a week), each pair of values was linearly connected. By assuming *RR*_*j*, ξ, *V*_ = *hR*_*j*, ξ, 0_ = 1 in Equation (14), the resulting death probability is equal to


(26) $$\begin{array}{l} p_{k}^{\left(Med_{k}\right)}=\sum_{j}p_{j,0,0}f_{k,j}^{\left(Med_{k}\right)}. \end{array}$$

By replacing the Equation (26) in the Equation (2), we estimated the number of infections ($\hat{N}$) from the beginning to the middle day of the ISTAT seroruvey (June 19, 2020; after 120 gg from the beginning) as


$$\begin{array}{l} \hat{N}=\overset{120}{\underset{k=1}{\sum }}\frac{D_{k}}{p_{k}^{\left(Med_{k}\right)}} \end{array}$$

and the ratio with the corresponding ISTAT estimate ($\bar{N}$) was used as correction factor of the Equation (26)


(27) $$\begin{array}{l} {\hat{p}}_{k}=\frac{\hat{N}}{\bar{N}}p_{k}^{\left(Med_{k}\right)}\text{with }\text{k}=1,...,120. \end{array}$$

#### Waves

In epidemiology, an internationally accepted definition of “wave” does not still exist; the term refers to the appearance of a plot of cases over time. In this paper, the word “wave” is used to indicate the part of the plot that lies between two local minima.

#### Health policy evaluation

The effects of applied health policies were determined by comparing the weekly and bi-weekly incidence rates before and after the day (*k*) they entered into force


$$\begin{array}{l} \frac{(N_{k+j}-N_{k})-(N_{k}-N_{k-j})}{N_{k}-N_{k-j}}\text{ with }k\in Z^{+}\text{ and }\text{j}=\;7,14. \end{array}$$

### Data cleaning methods

Dates of the ISS cumulative distribution of detected cases by age correlate with the most recent update. Those distributions were reordered to have non-decreasing functions. Since the VOC prevalence data in TESSEy cover a week, the data were linearly fitted to obtain daily prevalence. In addition, frequencies recorded before the official date of the first available samples were set to zero.

## Results

During the first 2 years of the COVID-19 pandemic (from February 24, 2020, to February 28, 2022; 736 days and 106 weeks) in Italy, there were 20,833,018 (20,728,922–20,937,373) infections and 152,358 deaths for an overall lethality rate of 0.73%. Health Institutions detected 63% of the total infections using 193,442,203 tests. From February 1, 2021, three VOCs became predominant: Alpha (February–June, 2021); Delta (July–December, 2021); and Omicron (January–February, 2022). Up to February 2022, 14.2% of people were unvaccinated (including children aged 0–4 years), 2.1% were waiting for the second shot, 12.2% had completed the basic vaccine cycle (one shot for the Jensen vaccine or two shots of other vaccines) > 4 months ago, 7.9% had completed the basic vaccine cycle in the last 4 months, and 63.6% had received the additional dose. Health policies evolved from a national lockdown to the development of a strong contact tracing system (the monthly number of tests increased from 506,496 in March 2020 to 17,089,550 in February 2022), accompanied by a large-scale vaccination campaign and additional local measures.

### Incidence curve

**Figure 1:**
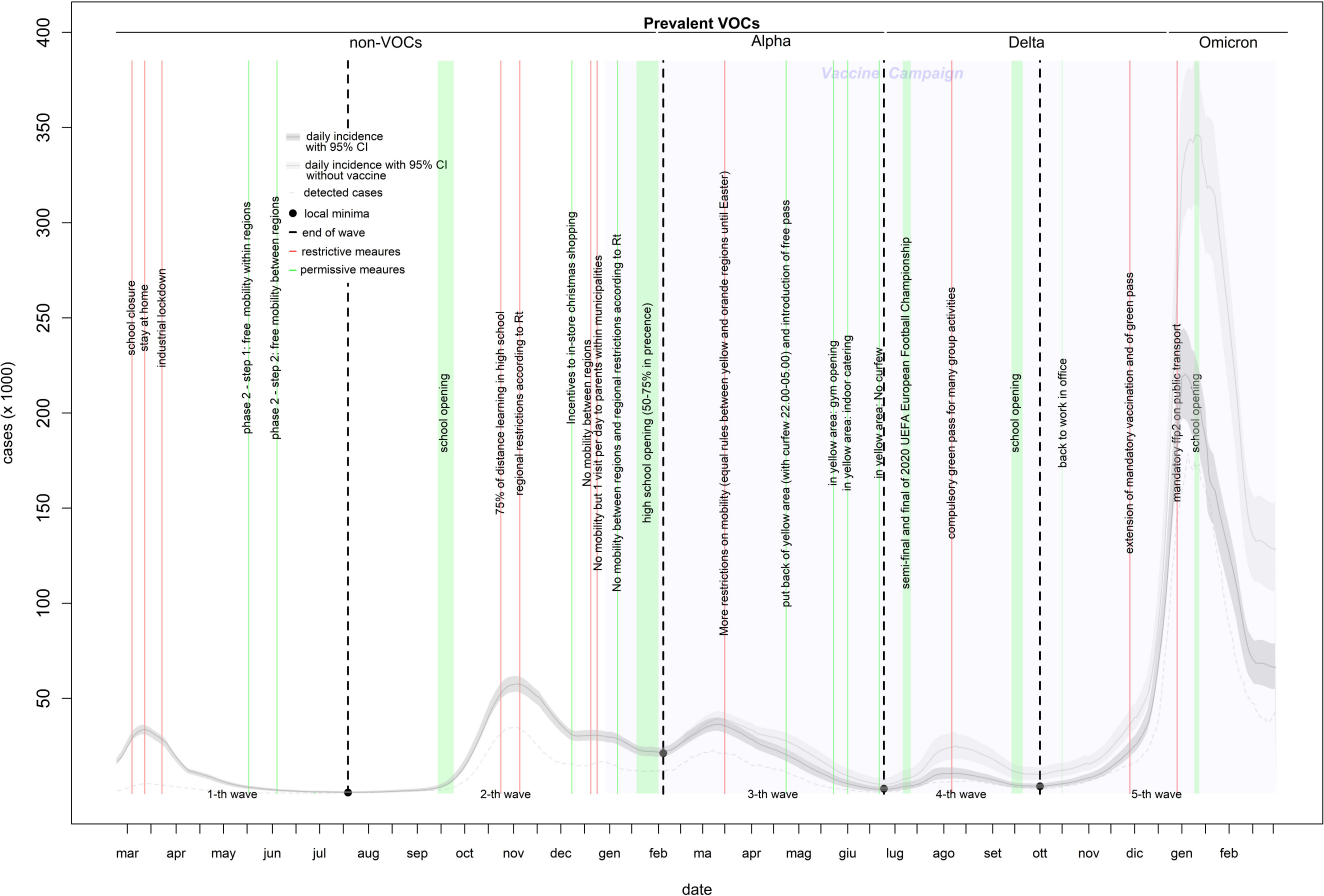
Daily incidence of COVID-19 in Italy (February 2020–2022).

The incidence curve in Figure 1 shows five waves. The first wave (characterized by infection with non-VOCs variants) lasted 147 days (from February 24 to July 19, 2020), included 1,526,561 infections (of which 240,802 were detected through 6,937,326 diagnostic tests), and peaked at 33,683 infections on March 12, 2020. The second wave (characterized by the predominance of the Alpha variant in the right tail) lasted 200 days (from July 20, 2020, to February 4, 2021), included 4,716,509 infections (of which 2,495,649 were detected through 29,891,933 diagnostic tests), and peaked at 57,594 infections on November 3, 2020. This wave also showed a hump of about 30,000 infections in the second half of December. The third wave (characterized by the Alpha variant) lasted 139 days (from February 5 to June 23, 2021), included 2,800,141 infections (of which 1,553,334 were detected through 37,736,565 diagnostic tests), and peaked at 36,471 infections on March 11, 2021. The fourth wave (characterized by the Delta variant) lasted 100 days (from June 24, 2021, to October 1, 2021), included 650,487 infections (of which 436,315 were detected through 23,693,891 diagnostic tests), and peaked at 10,617 infections on August 9, 2021. The fifth wave (characterized by a mixture of the Delta and Omicron variants) lasted 150 days (from October 2, 2021, to the end of the study period), included 11,139,320 infections (of which 8,344,165 were detected through 98,182,488 diagnostic tests), and peaked at 220,487 infections on January 1, 2022 (Table 1). From the third to the fifth wave, the proportion of infections among young individuals (0–19 years) increased by up to 30% (Supplementary Figure 1).

**Table 1:** Observed vs. expected (without vaccines) epidemiology of COVID-19 pandemic in Italy (February 2020–2022).

**Wave**	**Period**	**Observed pandemic**					**Expected pandemic without vaccine** ^*^			**Diagnosis**	**Test**
		**Deaths**	**Infection (95% CI)**	**Lethality (%)**	Max^**^	Day of max^**^	**Infection (95% CI)**	**Deaths**	**Lethality (%)**		
1	24/02/2020 19/07/2020	32,739	1,526,561 (1,542,962–1,510,247)	2.14	33,683	12/03/2020	1,526,561 (1,542,962–1,510,247)	32,739	2.14	240,802	6,937,326
2	20/07/2020 04/02/2021	62,595	4,716,509 (4,753,282–4679877)	1.33	57,594	03/11/2020	4,722,972 (4,759,726–4,686,359)	62,830	1.33	2,495,649	29,891,933
3	05/02/2021 23/06/2021	28,596	2,800,141 (2,832,521–2,767,945)	1.02	36,471	11/03/2021	3,369,666 (3,402,224–3,337,263)	40,844	1.21	1,553,334	37,736,565
4	24/06/2021 01/10/2021	3,620	650,487 (671,789–629,524)	0.56	10,617	09/08/2021	1,519,344 (1,540,474–1,498,358)	19,737	1.30	436,315	23,693,891
5	02/10/2021 28/02/2022	24,808	11,139,320 (11,278,202–11,001,288)	0.22	220,487	01/01/2022	19,135,912 (19,248,680–19,023,470)	110,298	0.58	8,344,165	98,182,488
Total	24/02/2020 28/02/2022	152,358	20,833,018 (20,728,922–20,937,373)	0.73	220,487	01/01/2022	30,274,455 (30,160,115–30,389,006)	266,448	0.88	13,070,266	196,442,203

* Vaccine campaign started at the end of the second wave (2020/12/27).

** Max = max number of daily infections.

### Lethality

**Figure 2:**
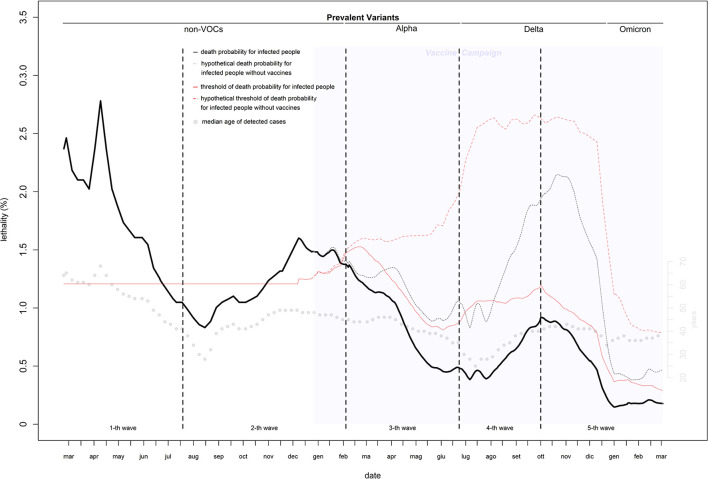
Daily lethality of COVID-19 in Italy (February 2020–2022).

**Figure 3:**
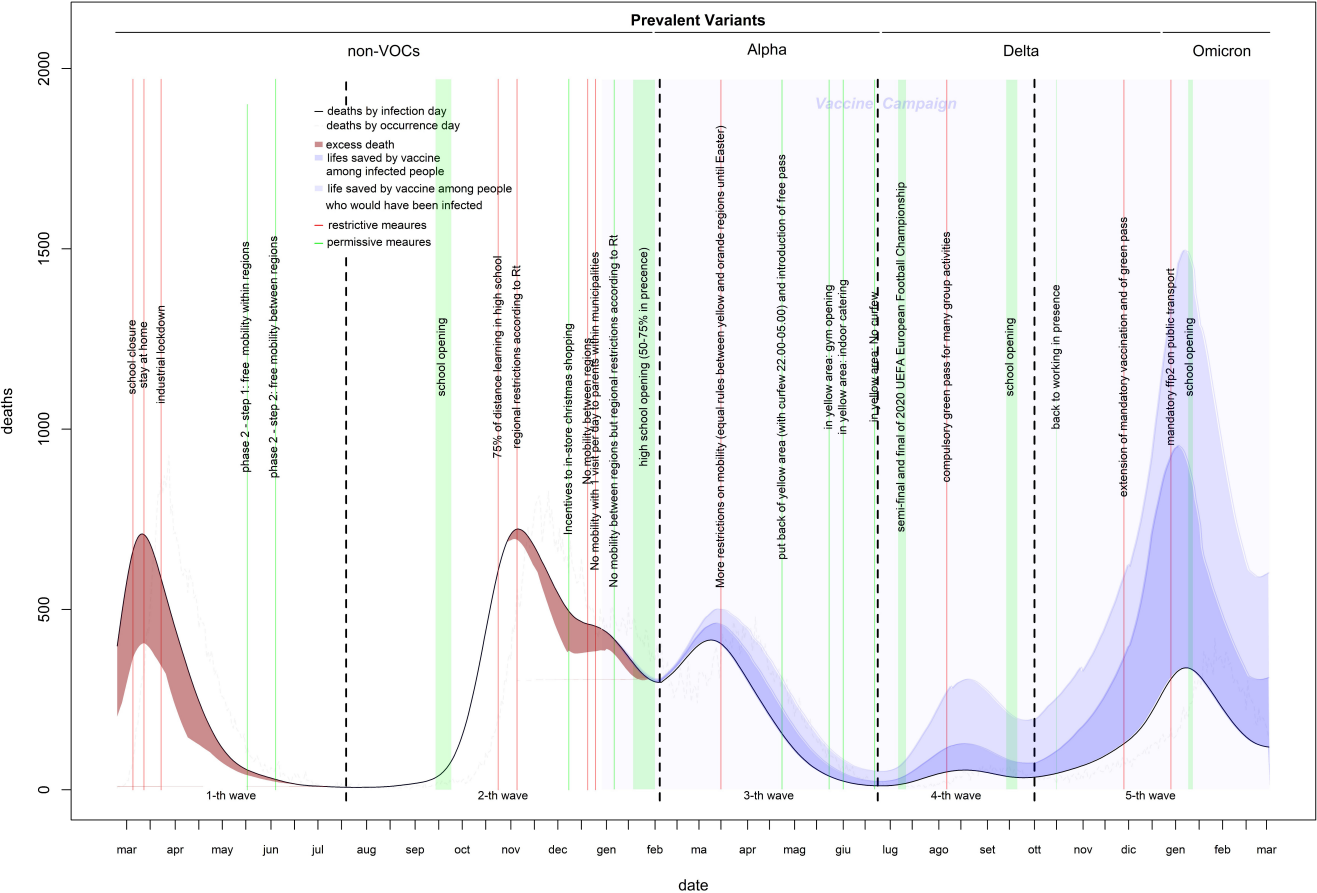
Daily deaths of COVID-19 in Italy (February 2020–2022).

Estimates of infection-related deaths during the 1st months of the pandemic were provided by a fictitious population (26) and adjusted using a correction factor (27) of 1.02%. Daily lethality ranged from 2.8% (first wave: April 9, 2020) to 0.15% (last wave: December 30, 2021), causing a total of 152,358 deaths with a peak of 723 deaths on November 5, 2020 (Figures 2, 3). After the peak, lethality in the first wave decreased to 1.0% on June 11, 2020, generating 32,739 deaths. Initially, lethality of the second wave, which caused 62,595 deaths, decreased to 0.8% on August 15, 2020, and then increased to a peak of 1.6% on December 8, 2020, then remained stable. The lethality in the third wave peaked at 1.4% on February 8, 2021, then decreased to 0.45% by June 8, 2021, and caused 28,596 deaths. The lethality in the fourth wave, which caused 3,620 deaths, initially decreased to 0.38% by July 6, 2021, and then increased to a peak of 0.91% by September 30, 2021. The lethality of the fifth wave, which caused 24,808 deaths, decreased continuously from a peak of 0.91% on October 2, 2021 to 0.15% on December 30, 2021, and then slowly increased to 0.18% by the end of February 2022. Two periods had the lethality higher than the threshold: the first 4 months of the first wave (March-June, 2020), with an excess of 14,134 deaths; and the last 3 months of the second wave (November, 2020–January, 2021), with an excess of 6,478 deaths (Figure 3).

### Impact of variants of concerns

Of five VOCs, three became prevalent (Alpha, Delta, and Omicron), each replacing prior variants at a faster pace. Omicron was responsible for the higher number of infections (7,557,368), while Alpha was associated with the largest number of deaths (29,167). Without vaccines, Delta would be the most virulent VOC with > 70,000 deaths (Supplementary Table 1).

### Vaccine effect

Vaccines reduced infections by 38% (from 25,045,987 to 15,604,551) and deaths by 62% (from 185,850 to 71,760). Of 114,090 lives saved, 62,902 (55%) would have resulted from infections prevented and 51,188 (45%) from the infections that occurred. Without vaccines, the expected number of infections would have been 30,274,455 (30,160,115–30,389,006), and the expected number of deaths 266,448 for a lethality of 0.88% (Figures 1–3 and Table 1).

### Health policies effects on estimated curves

The strongest restriction measures affecting all the population (initial stay at home, the November 2020 introduction of standardized prevention measures based geographic risk, and the Christmas 2020 and Easter 2021 restrictions) strongly reduced the curve rates (up to 1,000%) within a 1st week of their introduction. Industrial lockdown and specific restrictions (including 75% of high-school students who received distance learning) implemented in October 2020 are associated with smaller (from −37 to −227%) and slower (concentrated in the 2nd week) rate reductions. Curve rates increased after school openings (except after the last one of January 2022) and reductions in smart working, especially during the 2nd week. Rate increments after school openings reduced over time. In-shop Christmas 2020 incentives increased the incidence rate by 90–100% and the death rate by 45–60%. The introduction of a compulsory green pass reduced the rate of infection and death curve by 150–200 and 40–70%, respectively. The mandatory use of the FFp2 mask in closed places reduced the curve rates by 45–85% (Table 2). The introduction of rapid tests (from January 2021) increased the percentage of infections detected among children, particularly when schools were open (Supplementary Figure 1).

**Table 2:** Differences in rates of COVID-19 incidence and death curves before and after prevention measures.

**N**	**Date**	**Measures**	**Relative difference of rates (%)**			
			**Incidence curve**		**death curve**	
			**Weekly**	**Bi-weekly**	**Weekly**	**Bi-weekly**
1	05/03/2020	Schools closed	−67	−98	−73	−105
2	12/03/2020	Stop to all mobility—Stay at home	−189	−152	−238	−179
3	23/03/2020	Industrial lockdown	−77	−227	−77	−86
4	17/05/2020	Allowing intraregional mobility	30	48	35	57
5	04/06/2020	Free mobility	29	40	10	27
6	14/09/2020	Schools opened	609	1,128	544	1,027
7	24/10/2020	Several restrictions (including 75% DAD high school)	−57	−83	−37	−63
8	05/11/2020	Regional restrictions according to Rt	−508	−220	−151	−149
9	08/12/2020	Incentives for Christmas shopping	90	97	46	59
10	20/12/2020	No mobility between regions	−179	37	32	9
11	24/12/2020	Christmas rules: Just 1 visit per day to parents within municipalities	−643	−1,002	−62	−43
12	07/01/2021	Regional restrictions according to Rt	−182	−184	−33	−77
13	25/01/2021	High school opening (50–75% in presence)	124	175	123	150
14	15/03/2021	Easter rules: equal rules between yellow and orange regions	−458	−433	−239	−2,497
15	23/04/2021	Put back of yellow are (with curfew 22.00–05.00) and introduction of free pass	−21	−39	12	23
16	23/05/2021	In yellow area: Gym opening	15	33	28	50
17	01/06/2021	In yellow area: Indoor catering	29	47	33	56
18	21/06/2021	In yellow area: No curfew	118	167	94	133
19	09/07/2021	Semi-final and final of 2020 UEFA European Football Championship	38	257	188	497
20	06/08/2021	Compulsory green pass for many group activities	−208	−147	−41	−71
21	17/09/2021	Schools opened	90	97	94	120
22	15/10/2021	Back to work in office	24	85	27	66
23	27/11/2021	Extension of mandatory vaccination and of green pass	32	135	24	63
24	27/12/2021	Mandatory ffp2 on public transport	−55	−85	−44	−76
25	09/01/2022	Schools opened	−180	−1,524	−2,842	−309

Italy, February 2020–2022.

## Discussion

This paper provides a comprehensive picture for the first 2 years (February 2020–2022) of the COVID-19 pandemic in Italy, including the impact of VOCs, the vaccine campaign (until the third shot), and an evaluation of government health policies using only public data.

### Virus spread

Almost 40% of COVID-19 infections have gone undetected, likely because they were asymptomatic or paucisymptomatic. During the first wave, the virus primarily spread in the north of the country and was highly concentrated in the Lombardy region. The virus likely arrived in Italy through the airport system of Milan (the largest city in Lombardy), which includes one intercontinental and two international airports (one of which in Bergamo, the most hard-hit Italian city). Like other respiratory viruses, SARS-CoV-2 spreads directly or indirectly through person-to-person contact (especially in indoor environments) (29). Lombardy is the Italian region with the highest level of daily commuting for work or school^8^. Indeed, a study highlighted the association between the regional patterns of viral spread during the first wave and the origin-destination matrix of goods and food transportation and for the population (30). Another component responsible for the rapid spread of COVID-19 was unpreparedness. During the onset phase of the pandemic, hospitals followed WHO guidelines and tested only people with a known link to China (thus accelerating the spread of the virus). Fortunately, the quick stop to all national mobility on March 12, 2020, confined the virus to northern regions. Data collected during the second wave revealed similar patterns to those reported by other studies: the virus infects younger people first followed by those >70 years of age (26). Since retired people have fewer daily contacts than students and workers, who often use public transport and share indoor environments with others, it is likely that school and work transmission impacted the onset of the familial transmission chain among the elderly. Infected students and workers carried the virus home, transmitted the infection to other family members (31) and increased the probability that older relatives (including grandparents) would become infected, especially through presymptomatic or asymptomatic infections. During the summer months, those of 20–29 years of age were the hardest hit, presumably because of increased nightlife and other social activities. Without public health policies, it is likely that two waves would have occurred per year (similar to the fourth and fifth waves as shown in Figure 1): a winter wave (resulting from a higher number of transmissions from indirect contacts) and a summer wave (largely resulting from direct contacts). The former is longer (October to June), peaks in January, and is more virulent because it mainly involves families; the latter is shorter (July to September), peaks in August, and is less virulent because it primarily involves single people.

### Lethality

The estimates of lethality obtained by the fictitious populations (26) provided a cases count by June 20, 2020, that was very close to that estimated by the national ISTAT sero-survive^6^, with an error of 2%. This indicates that with a high median age of detected cases (with respect to that of the national population), the fictitious population provides reasonable estimates when the age distribution of cases is unknown. The virus's lethality was extremely high in the first 3 months of the pandemic, when the median age of detected cases was much higher than that of the population. This is the result of two serious errors: a lack of screening tests to reduce transmission from adult/young to elderly and the use of nursing homes to support hospitals with saturated capacity in the hardest hit regions (which increased infections among the most at risk population) (32). Lethality was higher than expected even during the second wave. The introduction of reliable rapid tests allowed a massive test campaign that kept lethality under the threshold from the right tail of the second wave. Lethality would have been under the threshold even without vaccines (Figure 2 dotted curves). Of the three prevalent VOCs in Italy, the first two were more virulent than the original strain, the third was not. To ensure that vaccines remain updated and new and potentially harmful variants are identified early, ongoing research on the evolution of virus genomes is crucial.

### Health policies

The stronger the restriction measure, the higher its efficacy and the more quickly it took effect. Supporting the assumption that the incidence curve was largely underestimated at the onset of pandemic, the initial lockdown impacted the death rate more than the infections rate. After the second wave, the large-scale screening helped to monitor the actual size of the outbreaks, especially among young students (often asymptomatic). Quarantining infected grandsons (tested at school) and parents (tested at work) likely protected the grandparents. This is supported by increased rates of infection and death curves after school openings (except the last one) and reductions in smart working. However, in schools, those increments declined over time until disappearing, presumably because school protocols became more and more effective. Even if the mandatory use of the ffp2 in the public transport reduced the curves rate by up to 70% during 2021 Christmas holidays, their true effect is shown after the schools opening, where rates drastically decreased of 1,500–2,800%. Although protective effect of the vaccines was reduced by the emergence of new variants, vaccination saved more than 110,000 lives and avoided the saturation of the health system without a need for stronger restriction measures even during periods of high virus circulation.

### Advice

It is necessary to monitor the evolution of SARS-CoV-2 in greater depth and to develop mathematical models that can predict future changes in its genome. A flexible pandemic plan able to adapt to the evidence of the data (collected through a digital and multi-connected surveillance system) should be developed through a multidisciplinary approach and shared with international health authorities. It should contain measures that are tailored for different combinations of virus transmissibility and virulence (from low-low to high-high). Estimates of territorial origin-destination matrices can help to simulate possible spatiotemporal patterns of virus spread. Initial settings of a public health response should refer to an “average” or “worst case” scenario and updates should follow data evidence.

## Data availability statement

Publicly available datasets were analyzed in this study. This data can be found at: https://github.com/floatingpurr/covid-19_sorveglianza_integrata_italia/tree/main/data.

## Ethics statement

Ethical review and approval was not required for the study on human participants in accordance with the local legislation and institutional requirements. Written informed consent from the participants' legal guardian/next of kin was not required to participate in this study in accordance with the national legislation and the institutional requirements.

## Author contributions

PF: study concept and design, acquisition of data, analysis and interpretation of data, drafting of the manuscript, critical revision of the manuscript for important intellectual content, and statistical analysis.

## Conflict of interest

The author declares that the research was conducted in the absence of any commercial or financial relationships that could be construed as a potential conflict of interest.

## Publisher's note

All claims expressed in this article are solely those of the authors and do not necessarily represent those of their affiliated organizations, or those of the publisher, the editors and the reviewers. Any product that may be evaluated in this article, or claim that may be made by its manufacturer, is not guaranteed or endorsed by the publisher.

## Footnotes

^1^https://covid19.who.int (accessed September 19, 2022).

^2^https://www.who.int/en/activities/tracking-SARS-CoV-2-variants (accessed September 19, 2022).

^3^https://github.com/italia/covid19-opendata-vaccini/tree/master/dati (accessed September 09, 2022).

^4^https://github.com/floatingpurr/covid-19_sorveglianza_integrata_italia/tree/main/data (accessed September 09, 2022).

^5^https://www.ecdc.europa.eu/en/publications-data/data-virus-variants-covid-19-eueea (accessed September 09, 2022).

^6^https://www.istat.it/it/archivio/242676 (accessed September 09, 2022).

^7^https://demo.istat.it (accessed September 09, 2022).

^8^ https://www.istat.it/it/archivio/139381
