# Supplementary figures and images for: The first 2 years of COVID-19 in Italy: Incidence, lethality, and health policies
Source: Front Public Health. 2022 Nov 1;10:986743. doi: 10.3389/fpubh.2022.986743 (PMC9664068; doi:10.3389/fpubh.2022.986743)

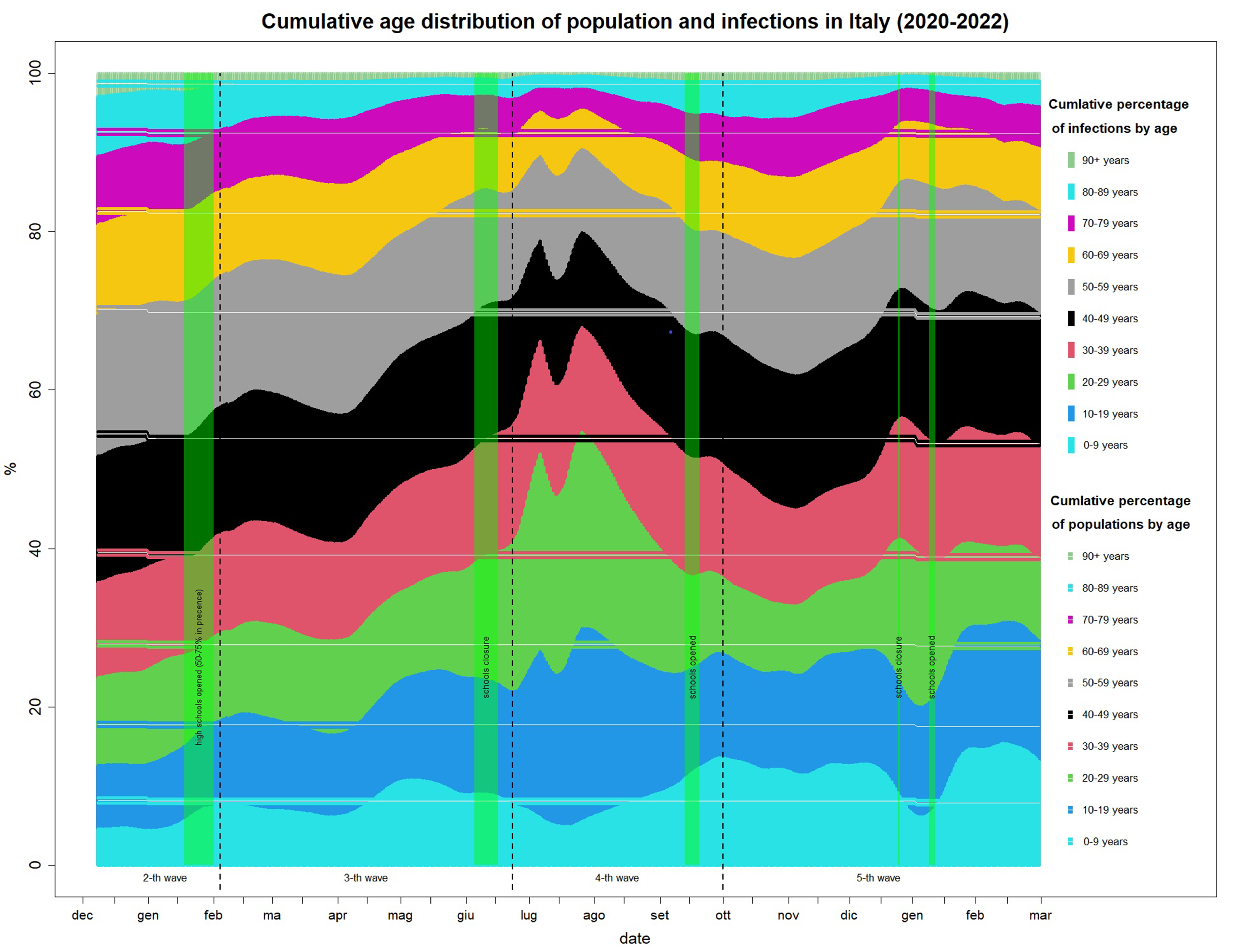

Supplement: Supplementary file 1 [file Image_1.jpg]
