# Supplementary material for: The first 2 years of COVID-19 in Italy: Incidence, lethality, and health policies
Source: Front Public Health. 2022 Nov 1;10:986743. doi: 10.3389/fpubh.2022.986743 (PMC9664068; doi:10.3389/fpubh.2022.986743)
Supplement: Supplementary file 2 [file Table_1.docx]

Table 1 SM: The epidemiology of COVID-19 by wave and variant of concern (VOC). Italy, February/2020-February/2022

| **Wave**  **(Period)** | **VOC** | **observed waves** | | |  | **expected withut vaccines waves** | | |
| --- | --- | --- | --- | --- | --- | --- | --- | --- |
|  |  | **Infections** | **deaths** | **lethality** |  | **Infections** | **deaths** | **lethality** |
| 1  24/02/2020 19/07/2020 | Alpha | 0 | 0 | 0.000 |  | 0 | 0 | 0.000 |
|  | Beta | 0 | 0 | 0.000 |  | 0 | 0 | 0.000 |
|  | Gamma | 0 | 0 | 0.000 |  | 0 | 0 | 0.000 |
|  | Delta | 0 | 0 | 0.000 |  | 0 | 0 | 0.000 |
|  | Omicron | 0 | 0 | 0.000 |  | 0 | 0 | 0.000 |
|  | Non-VOC | 1,526,561 | 32,739 | 0.021 |  | 1,526,561 | 32,739 | 0.021 |
| 2  20/07/2020 04/02/2021 | Alpha | 297,852 | 5,270 | 0.018 |  | 299,984 | 5,362 | 0.018 |
|  | Beta | 2,122 | 38 | 0.018 |  | 2,147 | 39 | 0.018 |
|  | Gamma | 19,362 | 260 | 0.013 |  | 19,548 | 266 | 0.014 |
|  | Delta | 11,200 | 354 | 0.032 |  | 11,226 | 356 | 0.032 |
|  | Omicron | 0 | 0 | 0.000 |  | 0 | 0 | 0.000 |
|  | Non-VOC | 4,385,973 | 56,673 | 0.013 |  | 4,390,067 | 56,807 | 0.013 |
| 3  05/02/2021 23/06/2021 | Alpha | 2,236,884 | 23,813 | 0.011 |  | 2,676,910 | 33,694 | 0.013 |
|  | Beta | 25,351 | 308 | 0.012 |  | 29,607 | 405 | 0.014 |
|  | Gamma | 183,590 | 1,440 | 0.008 |  | 231,033 | 2,171 | 0.009 |
|  | Delta | 40,664 | 376 | 0.009 |  | 70,603 | 1,084 | 0.015 |
|  | Omicron | 0 | 0 | 0.000 |  | 0 | 0 | 0.000 |
|  | Non-VOC | 313,652 | 2,659 | 0.008 |  | 361,513 | 3,490 | 0.010 |
| 4  24/06/2021 01/10/2021 | Alpha | 20,208 | 62 | 0.003 |  | 43,315 | 297 | 0.007 |
|  | Beta | 217 | 1 | 0.004 |  | 485 | 4 | 0.008 |
|  | Gamma | 4,098 | 10 | 0.002 |  | 8,741 | 47 | 0.005 |
|  | Delta | 577,957 | 3,418 | 0.006 |  | 1353,998 | 18,684 | 0.014 |
|  | Omicron | 0 | 0 | 0.000 |  | 0 | 0 | 0.000 |
|  | Non-VOC | 48,007 | 129 | 0.003 |  | 112,805 | 705 | 0.006 |
| 5  02/10/2021 28/02/2022 | Alpha | 5,359 | 22 | 0.004 |  | 9,561 | 113 | 0.012 |
|  | Beta | 503 | 2 | 0.003 |  | 814 | 7 | 0.008 |
|  | Gamma | 924 | 3 | 0.004 |  | 1,624 | 16 | 0.010 |
|  | Delta | 2,068,601 | 11,286 | 0.005 |  | 3,267,596 | 51,001 | 0.016 |
|  | Omicron | 7,557,368 | 9,908 | 0.001 |  | 13,352,469 | 44,037 | 0.003 |
|  | Non-VOC | 1,506,565 | 3,587 | 0.002 |  | 2,503,848 | 15,124 | 0.006 |
| All  24/02/2020  28/02/2022 | Alfa | 2,560,303 | 29,167 | 0.011 |  | 3,029,770 | 39,466 | 0.013 |
|  | Beta | 28,193 | 349 | 0.012 |  | 33,053 | 455 | 0.014 |
|  | Gamma | 207,974 | 1,713 | 0.008 |  | 260,946 | 2,500 | 0.010 |
|  | Delta | 2,698,422 | 15,434 | 0.006 |  | 4,703,423 | 71,125 | 0.015 |
|  | Omicron | 7,557,368 | 9,908 | 0.001 |  | 13,352,469 | 44,037 | 0.003 |
|  | Non-VOC | 7,780,758 | 95,787 | 0.012 |  | 8,894,794 | 108,865 | 0.012 |
|  | all | 20,833,018 | 152,358 | 0.007 |  | 3,027,455 | 266,448 | 0.009 |

*Vaccine campaign started at the end of the second wave (2020/12/27).
